# Data stream-pairwise bottleneck transformer for engagement estimation from video conversation

**DOI:** 10.3389/frai.2025.1516295

**Published:** 2025-06-27

**Authors:** Keita Suzuki, Nobukatsu Hojo, Kazutoshi Shinoda, Saki Mizuno, Ryo Masumura

**Affiliations:** NTT Human Informatics Laboratories, NTT Corporation, Yokosuka, Japan

**Keywords:** transformer, engagement, multiparty conversation, multimodal, classification, global token

## Abstract

This study aims to assess participant engagement in multiparty conversations using video and audio data. For this task, the interaction among numerous data streams, such as video and audio from multiple participants, should be modeled effectively, considering the redundancy of video and audio across frames. To efficiently model participant interactions while accounting for such redundancy, a previous study proposed inputting participant feature sequences into global token-based transformers, which constrain attention across feature sequences to pass through only a small set of internal units, allowing the model to focus on key information. However, this approach still faces the challenge of redundancy in participant-feature estimation based on standard cross-attention transformers, which can connect all frames across different modalities. To address this, we propose a joint model for interactions among all data streams using global token-based transformers, without distinguishing between cross-modal and cross-participant interactions. Experiments on the RoomReader corpus confirm that the proposed model outperforms previous models, achieving accuracy ranging from 0.720 to 0.763, weighted F1 scores from 0.733 to 0.771, and macro F1 scores from 0.236 to 0.277.

## 1 Introduction

Online meetings are essential tools for today's work environment, and the adoption of remote work has advanced due to the COVID-19 pandemic. Many companies continue to use hybrid work even after the pandemic. Therefore, the demand for online meetings has not decreased. However, unlike face-to-face meetings, online meetings present the challenge of enabling everyone to stay focused due to a diminished sense of participation. In such online environments, it can be difficult to capture nonverbal cues such as gaze, facial expressions, and tone of voice, leading to an increased risk of declines in engagement (Sukumaran and Manoharan, 2024). Therefore, it has become important to continually estimate the engagement of online meeting participants and provide appropriate feedback.

Engagement estimation has evolved from analyzing individual behaviors to modeling complex group interactions across various modalities. Early work focused on single-person unimodal signals, such as facial expressions or gaze (Savchenko et al., 2022; Singh et al., 2023), followed by multimodal approaches that combine audio and video to improve robustness (Pan et al., 2023; Kumar et al., 2024). Dyadic settings introduced the importance of interpersonal cues (Dermouche and Pelachaud, 2019; Chen et al., 2022). Recent work has expanded to multiparty scenarios using only visual data (Lee et al., 2023). However, as shown in recent studies (Kim et al., 2023; Suzuki et al., 2024), modeling both audio and visual signals across participants improves engagement prediction. We therefore address *multiparty multimodal engagement estimation*, defined as estimating a target participant's engagement from the audiovisual data of all participants, which remains a challenging and underexplored area.

**Figure 1:**
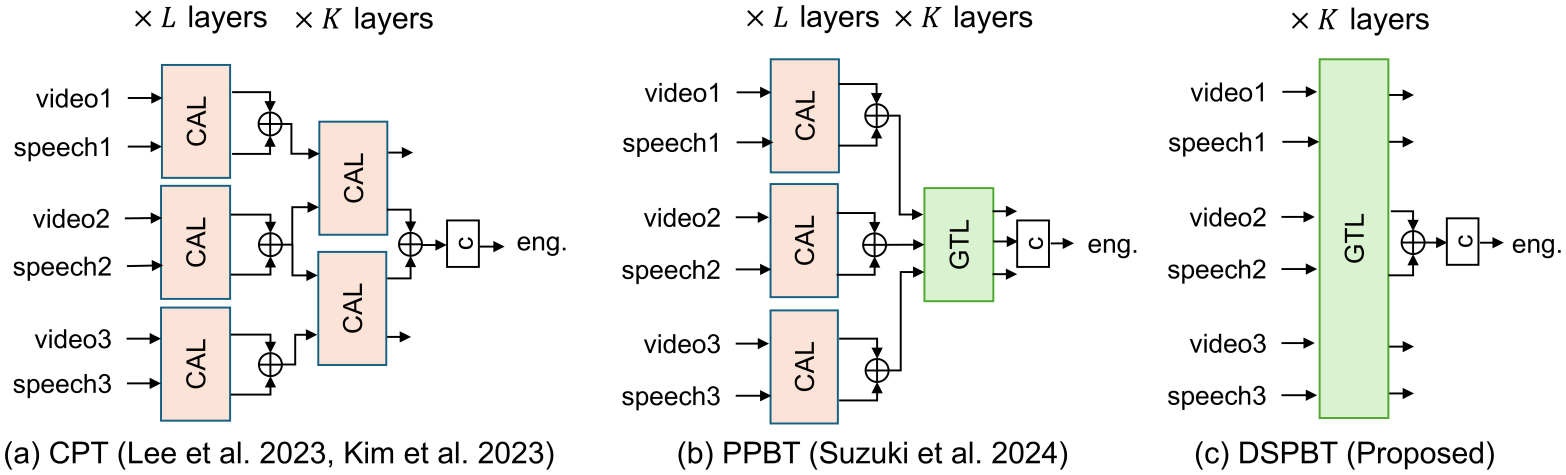
Overview of previous and proposed models. “CAL” and “GTL” respectively denote cross-attention and global-token layers in Figure 2. “c” denotes classification layer. Each figure illustrates case in which number of participants *N* is 3 and target participant ID n^*^ is 2. **(a)** CPT (Lee et al., 2023; Kim et al., 2023). **(b)** PPBT (Suzuki et al., 2024). **(c)** DSPBT (Proposed).

Figure 1 illustrates the existing and proposed multiparty multimodal engagement estimation models. A central challenge in multiparty multimodal engagement estimation lies in modeling interactions among multiple data streams—namely, different modalities (e.g., video and audio) from multiple participants. To make accurate predictions for a target participant, the model used must effectively capture both cross-modal interactions within each participant and cross-person interactions across participants. Previous studies have addressed this by introducing cross-person transformers (CPTs) (Lee et al., 2023; Kim et al., 2023), which rely on hierarchical combinations of cross-attention layers to separately model these two types of interaction. More recently, global token-based architectures have been proposed as a more efficient alternative for representing interactions between high-dimensional streams, avoiding the combinatorial explosion of direct attention (Sun et al., 2023; Nagrani et al., 2021). In the context of engagement estimation, participant-pairwise global tokens have shown promise in modeling cross-person interactions more effectively (Suzuki et al., 2024). However, the previous model still depends on cross-attention mechanisms for cross-modal fusion, leaving challenges related to redundancy and scalability unresolved. In this work, we address this limitation by extending global token-based modeling to unify both cross-modal and cross-person interactions within a single architecture. Our proposed model introduces a data stream-pairwise structure that enables efficient and accurate engagement estimation across all modalities and participants in multiparty conversations.

To thoroughly investigate this question in a realistic multiparty environment, we require a dataset that meets four key criteria: it should (1) include engagement annotations, (2) contain both video and audio data, (3) capture multiparty interactions, and (4) ideally be publicly available. To the best of our knowledge, only the RoomReader corpus (Reverdy et al., 2022) satisfies all these requirements simultaneously (see Section 2.4 and Table 1). RoomReader provides over 8 h of online multiparty conversations with synchronized video and audio, along with annotations of each participant's engagement level, making it uniquely suited to this research.

**Table 1:** Representative engagement estimation datasets: comparison of participants, modalities, and availability.

**Dataset**	**Participants**	**Modalities**	**Availability**
DAiSEE (Gupta et al., 2016)	Single	Video	Public
EmotiW 2018 (Dhall et al., 2018)	Single	Video	Restricted
EmotiW 2019 (Dhall et al., 2019)	Single	Video	Restricted
NoXi (Cafaro et al., 2017)	Dyadic	Video, Audio	Public
RECOLA (Ringeval et al., 2013)	Dyadic	Video, Audio, Physiological	Public
DAMI-P2C (Chen et al., 2022)	Dyadic	Video, Audio	Public
RoomReader (Reverdy et al., 2022)	Multiparty	Video, Audio	Public

While RoomReader provides behavior-based engagement annotations suitable for real-time modeling, it is important to contrast this with more traditional definitions and measurement approaches. Engagement is often defined as a multi-dimensional construct encompassing behavioral, emotional, cognitive, and agentic components and is typically measured via self-reports (Fredricks et al., 2004, 2016; Sinatra et al., 2015). However, such labels lack temporal granularity and may diverge from observable behavior due to annotation bias. These limitations make them ill-suited for the application assumed in this study, which requires not only real-time inference and feedback during conversations but also engagement labels that align consistently with audiovisual input–since misalignment can cause models to learn spurious or non-generalizable associations. We therefore adopt the RoomReader corpus (Reverdy et al., 2022), which provides temporally dense, behavior-based annotations that are well-suited for multimodal modeling.

The contributions of this research are as follows.

- **Introduction of global tokens that handle interactions among data streams, i.e., multiple modalities from multiple participants in conversations:** To estimate engagement in multiparty conversations, we introduce global tokens to manage the interactions among multiple input data streams, enabling efficient modeling.
- **Proof of effect:** By introducing global tokens that manage interactions between data streams, we demonstrate that higher accuracy can be achieved through engagement-estimation experiments on the publicly available RoomReader corpus compared with previous methods.

These contributions provide new directions for estimating multi-participant engagement.

This article is structured as follows. Section 2 reviews the previous research on engagement estimation, small-group interaction modeling, transformer-based multimodal approaches, and relevant corpora. Section 3 details the baseline and our proposed method. Section 4 explains the experimental setup and datasets, and Section 5 reports and discusses the experimental results. Finally, Section 6 concludes the paper and suggests possible future directions.

## 2 Related works

Research on automatic engagement recognition has evolved from early work on individual behaviors to more complex modeling of social interaction. In this section, we briefly review approaches that estimate engagement of individual participants before shifting to methods that incorporate interpersonal group-level dynamics (see Table 2).

**Table 2:** Representative engagement estimation methods: comparison of study, dataset, participants, modality and model.

**References**	**Dataset**	**Participants**	**Modality**	**Model**
Li and Hung (2019)	EmotiW 2018	Single	Video	CNN + LSTM
Wang et al. (2019)	EmotiW 2019	Single	Video	LSTM ensemble of regressors
Huynh et al. (2019)	EmotiW 2019	Single	Video	LSTM ensemble of regressors
Ma et al. (2021)	DAiSEE	Single	Video	Bi-LSTM
Kim et al. (2023)	DAMI-P2C	Dyadic	Video, Audio	CNN + Transformer
Xiong et al. (2023)	Original online learning videos	Multiparty	Video	CNN + Transformer
Lee et al. (2023)	RoomReader	Multiparty	Video, Audio	CNN + Transformer
Suzuki et al. (2024)	RoomReader	Multiparty	Video, Audio	CNN + Transformer

### 2.1 Engagement estimation

Early research on automatic engagement recognition spans diverse contexts, including education, social robotics, and conversational interfaces. Rich et al. (2010) pioneered engagement recognition in human–robot interaction by using backchannel cues. Others leveraged nonverbal signals: for instance, Bednarik et al. (2012) used gaze patterns to recognize engagement in group conversations, and Sanghvi et al. (2011) analyzed body posture features to estimate child engagement with a robot tutor. In educational settings, detecting student engagement from face and body cues has been a major focus (Grafsgaard et al., 2014).

Previous studies estimated engagement using the convolutional neural network-long short-term memory (CNN-LSTM) and CNN-transformer on the basis of the video and speech data of the target participant (Li and Hung, 2019; Xiong et al., 2023). Engagement estimation has advanced with the introduction of models such as the bootstrap model ensemble (BOOT) and the ensemble model (ENS-MODEL), which have facilitated the use of bootstrapping and ensembling methods (Wang et al., 2019; Huynh et al., 2019). Wang et al. (2019) introduced a model ensemble with a rank-based loss function for engagement intensity regression. By aggregating multiple models, their approach achieved a top rank in the EmotiW 2019 engagement challenge (mean squared error of 0.0626 on the test set). Similarly, Huynh et al. (2019) developed an ensemble-based regression using facial behavior features such as action units and head motion, demonstrating high-level performance in the same challenge. These ensemble models mitigate individual model biases and variance, leading to more robust engagement estimation.

Additionally, hierarchical temporal multi-instance learning (HTMIL) uses a bidirectional long short-term memory (Bi-LSTM) with multi-scale attention to achieve both clip-level and video-level objectives, effectively capturing short- and long-term patterns (e.g., momentary distraction vs. sustained attention) by splitting a video into temporal segments, as proposed in Ma et al. (2021).

These methods primarily focus on estimating the engagement of an individual participant based on unimodal or multimodal signals, without modeling interactions among participants. In contrast, our work considers engagement as a phenomenon that emerges through social interaction, particularly in multiparty settings. We therefore turn next to models that explicitly handle small-group interactions.

### 2.2 Small-group interaction models

Graphical models have been instrumental in analyzing interactions, notably for group-performance prediction, behavior recognition, social-field modeling, and interaction recognition (Lin and Lee, 2020; Yang et al., 2020; Zhou et al., 2019; Li et al., 2020). However, in the context of larger, multi-participant settings, the effectiveness of graphical models suffers. The complication arises from the scalability of these models and the added complexity of interactions that come with larger group sizes. The web of intricate interactions in such settings introduces a level of complexity that conventional graphical models cannot handle; the nuances of complex group dynamics prove difficult to accurately capture. It seems rather that more sophisticated approaches are needed to handle the diverse and dynamic nature of interactions in multi-participant environments. Recently, transformer-based methods that employ attention mechanisms among participants have been proposed, offering new possibilities for modeling interactions more effectively in large-scale social settings (Lee et al., 2023; Kim et al., 2023; Suzuki et al., 2024).

### 2.3 Transformer modeling by using global tokens

There are studies that take a transformer-based approach to representing interactions between modalities using global tokens for multimodal sentiment analysis (Sun et al., 2023; Nagrani et al., 2021). These studies embrace the novel approach of using interactions based on global tokens to help make the process much more efficient. Global tokens are abstract representations of information that are shared across the modalities, so they can exchange info, such as video and audio, without resorting to the attention mechanism. They incur lower computational costs and information redundancy than the attention mechanism. It has also been reported that the use of global tokens facilitates the integration of information across different modalities, resulting in overall models with higher accuracy. Sentiment analysis generally aims to identify or categorize emotional states (e.g., positive or negative feelings) based on audiovisual data. In contrast, engagement analysis focuses on the level of participation of meeting attendees, which differs from emotional analysis. Nevertheless, the global-token approach remains relevant for both sentiment and engagement tasks because it offers an efficient way to capture cross-modal relationships by acting as an attention bottleneck. In this work, we build on previous research involving global tokens but shift our emphasis from sentiment analysis to engagement estimation, centering on how actively participants are involved in the conversation rather than on their emotional valence. There are studies that focused on multi-participant meetings in engagement estimation, where global tokens are used to represent interactions in part of the model: their effectiveness has been demonstrated (Suzuki et al., 2024).

### 2.4 Corpus for engagement estimation

In Table 1, we compare representative datasets commonly used for engagement estimation. Key attributes are summarized, including the participant setup (dyadic or multiparty), recorded modalities, and data availability. Notably, only the RoomReader dataset provides publicly available audiovisual data of multiparty interactions, which is why our work utilizes RoomReader exclusively (Reverdy et al., 2022). Other datasets either focus on dyadic interactions or are not publicly released or lack combined video and audio modalities. The primary uses for engagement estimation in human–computer-interaction research have been the Remote Collaborative and Affective Interaction (RECOLA) and Nonverbal Interaction in Expert–Novice Interaction (NoXi) corpora (Ringeval et al., 2013; Cafaro et al., 2017). These corpora have played an important role in the field, recording dyadic interactions through detailed recordings of conversational exchanges between two participants. In particular, as shown in Table 1, existing engagement datasets vary widely, but early works often focused on dyadic interactions: for example, the NoXi corpus captures two-person video chats—specifically remote expert–novice conversations (Cafaro et al., 2017). Meanwhile, the RECOLA corpus contains remote dyadic collaborations featuring rich modalities (audio, video, ECG, and EDA) that include engagement (Ringeval et al., 2013). Consequently, the RECOLA and NoXi corpora serve as essential research resources for researchers seeking to understand the subtle variations in engagement between two individuals.

Some datasets focus on parent–child scenarios: Dyadic Affect in Multimodal Interaction—Parent to Child (DAMI-P2C) records in-lab story-reading sessions (dyads), annotated for child engagement and parent–child relationship measures (e.g., attachment, relational frustration, and parenting stress), among others (Chen et al., 2022). There are also “in-the-wild” single-participant datasets: the Emotion Recognition in the Wild 2018 and 2019 challenges (EmotiW 2018 and 2019) introduced webcam videos of students watching MOOC lectures, annotated for engagement (Dhall et al., 2018, 2019). Meanwhile, the Dataset for Affective States in E-Environments (DAiSEE) captures user engagement in online learning contexts (Gupta et al., 2016), alongside other affective states such as boredom, confusion, and frustration. However, these datasets are not multiparty (involving only one learner) and were only released for competition use.

Our research goes beyond the scope of dyadic engagement estimation by introducing the RoomReader corpus to understand engagement in multiparty conversational settings, a method also used in a previous study because fewer datasets feature multiparty (group) interactions (Lee et al., 2023). Therefore, to enable research on engaged group conversations with reproducible results, we focus on the RoomReader corpus in our study. RoomReader uniquely offers a public, multimodal, group-interaction dataset containing over 8 h of online multiparty conversations with synchronized video and audio, along with student engagement annotations. This combination of group interaction, audio-visual modality, and public accessibility is exclusive to RoomReader among current datasets, making it essential for our multiparty engagement research.

## 3 Method

### 3.1 Cross-attention layer and global token layer for data stream interaction modeling

**Figure 2:**
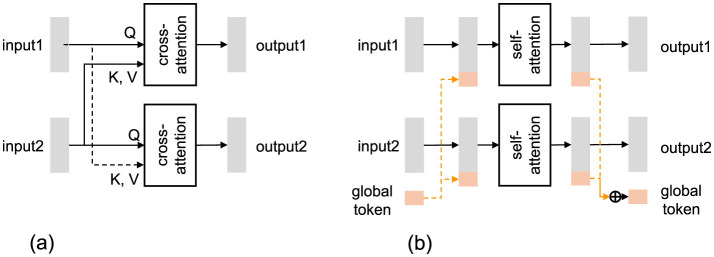
Illustration of interaction models between two data streams. “Cross-attention” and “self-attention” denote cross-attention and self-attention transformer-encoder layers, respectively. **(a)** Cross-attention layer (CAL). **(b)** Global token layer (GTL).

To model interactions among multiple data streams–that is, different modalities (e.g., audio and video) across multiple participants–previous work has proposed various mechanisms, notably the Cross-Attention Layer (CAL) and the Global Token Layer (GTL) (Figure 2). While CAL has been widely adopted in recent approaches to handle pairwise stream interactions, our proposed architecture adopts a unified modeling approach based on GTL, which enables more efficient and scalable fusion across modalities and participants. When more than two input streams are involved, GTL can be implemented in two forms: the common GTL, which aggregates all streams into shared tokens, and the pairwise GTL, which processes each pair of streams individually through a shared bottleneck (Suzuki et al., 2024). In this section, we present the formal definitions of CAL, GTL (common), and GTL (pairwise) to clarify their functional differences and modeling characteristics. We begin by introducing the notation for input data streams and then describe the mathematical formulation of each layer.

#### 3.1.1 Cross-attention layer

The Cross-Attentional Layer (CAL) (Figure 2a) takes two input data streams, ***X***_1_ and ***X***_2_, and models their interaction to output two corresponding streams, ***Y***_1_ and ***Y***_2_. The inputs and outputs are matrices of shape *D* × *T*_1_ for ***X***_1_, ***Y***_1_ and *D* × *T*_2_ for ***X***_2_, ***Y***_2_, where *D* is the feature dimension and *T*_1_ and *T*_2_ are the time lengths.

CAL is implemented by repeating a cross-attention transformer layer (Vaswani et al., 2017) *L* times. Let $H_{1}^{0}=X_{1}$ and $H_{2}^{0}=X_{2}$ denote the initial inputs. Then, for each layer *ℓ* = 1, …, *L*, the intermediate representations are computed as follows:


(1) $$\begin{array}{l} H_{1}^{\ell }=\text{CrossAttention}\left(H_{1}^{0},H_{2}^{\ell -1},H_{2}^{\ell -1};\theta_{1}^{\ell }\right), \end{array}$$

(2) $$\begin{array}{l} H_{2}^{\ell }=\text{CrossAttention}\left(H_{2}^{0},H_{1}^{\ell -1},H_{1}^{\ell -1};\theta_{2}^{\ell }\right). \end{array}$$

Here, CrossAttention(***Q***, ***K***, ***V***; θ) denotes a cross-attention transformer layer, where ***Q***, ***K***, and ***V*** represent the query, key, and value matrices, respectively. $\theta_{1}^{\ell }$ and $\theta_{2}^{\ell }$ are the parameters of each respective transformer at layer *ℓ*.

The final outputs of CAL are defined as follows:


(3) $$\begin{array}{l} Y_{1}=H_{1}^{L},\;Y_{2}=H_{2}^{L}. \end{array}$$

In this study, we define CAL layers as a function mapping from ***X***_1_ and ***X***_2_ to ***Y***_1_ and ***Y***_2_ using shared notation:


(4) $$\begin{array}{l} Y_{1},Y_{2}=CAL\left(X_{1},X_{2};\theta_{\text{CAL}}\right). \end{array}$$

Here, $\theta_{\text{CAL}}={\left\{\theta_{1}^{\ell },\theta_{2}^{\ell }\right\}}_{\ell }$ represents the set of trainable parameters used in the CAL layers.

#### 3.1.2 Global token layer (common)

**Figure 3:**
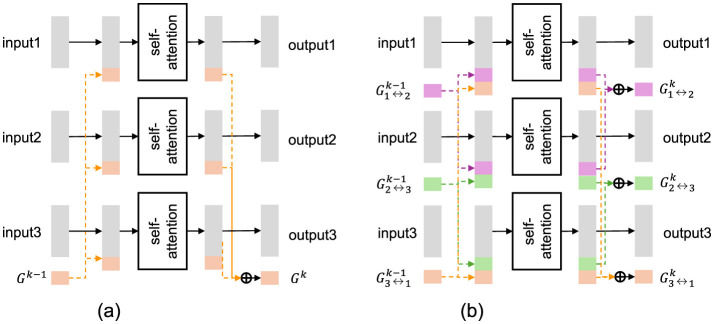
Illustration of common and pairwise GTL when there are three input data streams. **(a)** GTL (common). **(b)** GTL (pairwise).

By contrast, the common GTL (Figure 3a) forces all data streams to exchange information through a set of trainable tokens, ***G***, greatly reducing potential redundancy across frames. GTL takes *N*(≥ 2) input data streams ***X***_1_, …, ***X***_*N*_ and models their interactions via ***G***. It outputs the corresponding data streams and updated global tokens for use in subsequent layers. Each stream ***X***_*n*_ and ***Y***_*n*_ is a matrix in ${\mathbb{R}}^{D\times T_{n}}$, and the global tokens are represented as a matrix ***G*** ∈ ℝ^*D*×*B*^, where *B* represents the dimension and length of the bottleneck tokens.

GTL is implemented by repeating a transformer encoder with self-attention for *L* layers. Let $H_{n}^{0}=X_{n}$ and ***G***^0^ denote the initial inputs. At each layer *ℓ* = 1, …, *L*, the intermediate representations are computed as follows:


(5) $$\begin{array}{l} \left[H_{n}^{\ell }∥G_{n}^{\ell }\right]=TransformerEnc\left(\left[H_{n}^{\ell -1}∥G^{\ell -1}\right];\theta_{n}^{\ell }\right), \end{array}$$

where [·||·] denotes concatenation along the temporal (sequence) dimension. The global token is updated by aggregating across all *n* as follows:


(6) $$\begin{array}{l} G^{\ell }=\underset{n}{\sum }G_{n}^{\ell }. \end{array}$$

The final outputs of the GTL are defined as follows:


(7) $$\begin{array}{l} Y_{n}=H_{n}^{L}. \end{array}$$

In this study, we define the GTL module as follows:


(8) $$\begin{array}{l} Y_{1},\ldots ,Y_{N}={GTL}_{common}\left(X_{1},\ldots ,X_{N};\theta_{\text{GTLc}}\right). \end{array}$$

Here, TransformerEnc(·;θ) denotes a self-attention-based Transformer Encoder layer, and $\theta_{\text{GTLc}}=\left\{{\left\{\theta_{n}^{\ell }\right\}}_{\ell ,n},G^{0}\right\}$ represents the parameters used in the GTL module.

The advantage of GTL is that interactions between different modalities or participants are channeled through a compact set of tokens, potentially alleviating computational bottlenecks and improving the learning of cross-modal relationships. On the other hand, CAL can suffer from higher complexity when dealing with longer input sequences, particularly those exhibiting high redundancy across frames, such as audio or video.

#### 3.1.3 Global token layer (pairwise)

To accurately model the interaction between each pair of data streams, the previous study proposed defining multiple global token sequences, each corresponding to a pair of data streams (Suzuki et al., 2024). The pairwise Global Token Layer (GTL) (Figure 3b) also takes *N* (*N* ≥ 2) input data streams ***X***_1_, …, ***X***_*N*_ and models their interactions, producing the corresponding output streams ***Y***_1_, …, ***Y***_*N*_, in a manner similar to the common GTL.

However, it differs from the common GTL in that it defines and utilizes multiple global token sequences ***G***_*m* ↔ *n*_ to capture pairwise interactions among the input streams, *m* and *n*. Each token sequence $G_{m↔n}\in {\mathbb{R}}^{D\times B}$ is dedicated to modeling the interaction between ***X***_*m*_ and ***X***_*n*_.

Let $H_{n}^{0}=X_{n}$ and $G_{m↔n}^{0}=G^{0}$ denote the initial input and pairwise tokens. Then, at each layer *ℓ* = 1, …, *L*, we compute:


(9) $$\begin{array}{l} \left[H_{m}^{\ell }∥\underset{n\in J\backslash m}{⊕}G_{m\to n}^{\ell }\right]=TransformerEnc\left(\left[H_{m}^{\ell -1}∥\underset{n\in J\backslash m}{⊕}G_{m↔n}^{\ell -1}\right];\theta_{m}^{\ell -1}\right), \end{array}$$

where ⊕ represents vector concatenation (⊕_*n* = 1, 2_ *A*_*n*_ = [*A*_1_||*A*_2_]), and $\theta_{m}^{\ell -1}$ denotes the parameters of the *l*-th layer of the Transformer-encoder block for the *m*-th data stream.

Instead of Equation 6 in common GTL, each global-token sequence is updated by summing the variables that represent the dependencies of participants in both directions as follows:


(10) $$\begin{array}{l} G_{m↔n}^{\ell }=G_{m\to n}^{\ell }+G_{n\to m}^{\ell } \end{array}$$

(11) $$\begin{array}{l} G_{n↔m}^{\ell }=G_{n\to m}^{\ell }+G_{m\to n}^{\ell }. \end{array}$$

The final outputs of the pairwise GTL are defined as follows:


(12) $$\begin{array}{l} Y_{n}=H_{n}^{L}, \end{array}$$

We define the pairwise GTL function as follows:


(13) $$\begin{array}{l} Y_{1},\ldots ,Y_{N}={GTL}_{pairwise}\left(X_{1},\ldots ,X_{N};\theta_{\text{GTLp}}\right). \end{array}$$

Here, $\theta_{\text{GTLp}}=\left\{{\left\{\theta_{n}^{\ell }\right\}}_{n,\ell },G^{0}\right\}$ represents the parameters of the pairwise GTL module.

### 3.2 Task

**Figure 4:**
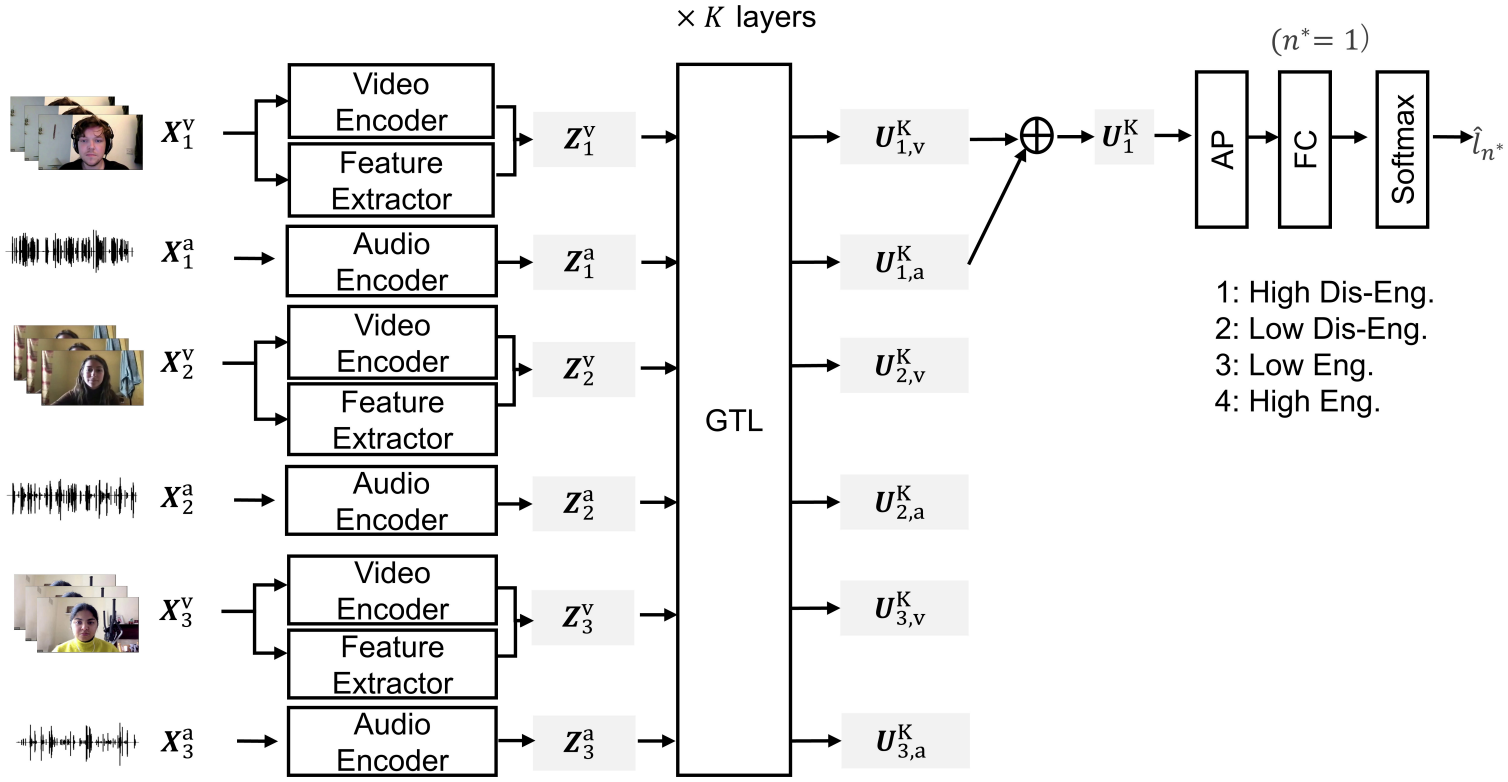
Schematic diagram of engagement-estimation task, DSPBT. “AP” and “FC” denote attention pooling and fully connected layer, respectively.

We adopt the four engagement classes for training and evaluation, as in Multipar-T, as a baseline method (Lee et al., 2023). This four-label design is consistent with other engagement corpora (e.g., DAiSEE) and has effectively captured varying degrees of participant attentiveness and dis-engagement. Figure 4 provides an outline of the engagement-estimation task. Let *N* represent the total number of participants involved in a conversation. Our goal is to estimate the engagement of a specific participant n^*^ ∈ {1, ⋯ , *N*} at a certain time *t* based on conversation data. More specifically, we estimate engagement over the time interval [*t* − *D, t*], meaning that a video and audio segment with a duration of *D* s serves as the context. In the remainder of this section, we omit the explicit mention of time *t* and refer to the video and audio clips of participant *n* as $X_{n}^{\text{v}}$ and $X_{n}^{\text{a}}$, respectively. We also denote the engagement label of the target participant as $l_{n^{*}}$. We framed the task as a four-class classification problem, where *l*_*n*_ ∈ {1 (High Dis-Engagement), 2 (Low Dis-Engagement), 3 (Low Engagement), 4 (High Engagement)}. The input data ***X*** are represented as follows:


(14) $$\begin{array}{l} X=\left\{X_{1}^{\text{v}},X_{1}^{\text{a}},\cdots \;,X_{N}^{\text{v}},X_{N}^{\text{a}}\right\}, \end{array}$$

Thus, the engagement-estimation task can be formulated as follows:


(15) $$\begin{array}{l} {\hat{l}}_{n^{*}}=f\left(X,n^{*};\Theta \right), \end{array}$$

where *f*(·) is the classification function provided by the model, and Θ denotes the parameters of that model. This setup follows the approach in a previous study (Lee et al., 2023), with the addition of audio data streams.

### 3.3 Multi-person bottleneck transformers

#### 3.3.1 Baseline multi-person bottleneck transformer

The baseline multi-person bottleneck transformer (MPBT) model (Suzuki et al., 2024) uses CALs for the cross-modal interaction model and GTLs for the cross-person interaction model. It initially uses pre-trained encoders to extract audio and video features from the input data for each participant *n* ∈ {1, ⋯ , *N*} through speech and video encoders as follows:


(16) $$\begin{array}{l} Z_{n}^{\text{a}}=SpeechEncoder\left(X_{n}^{\text{a}};\theta_{\text{a}}\right), \end{array}$$

(17) $$\begin{array}{l} Z_{n}^{\text{venc}}=VideoEncoder\left(X_{n}^{\text{v}};\theta_{\text{venc}}\right), \end{array}$$

where VideoEncoder(·) and SpeechEncoder(·) are functions that project data into feature vectors for video and speech, respectively. The parameters of the encoders are denoted as θ_venc_ and θ_a_, and $Z_{n}^{m}\in {\mathbb{R}}^{D_{\text{model}}\times T_{m}}$ represents the feature vectors for the modality *m* ∈ {venc, a}, where *D*_model_ is the feature dimension and *T*_*m*_ is the time length. Similar to Multipar-T (Lee et al., 2023), we also extract features, such as head movements, from the video using OpenFace (Baltrušaitis et al., 2016), which are then added to the output of the video encoder as follows:


(18) $$\begin{array}{l} Z_{n}^{\text{vfeat}}=VideoFeatureExtractor\left(X_{n}^{\text{v}};\theta_{\text{vfeat}}\right), \end{array}$$

(19) $$\begin{array}{l} Z_{n}^{\text{v}}=Z_{n}^{\text{venc}}+FC\left(Z_{n}^{\text{vfeat}};\theta_{\text{F}{\text{C}}_{1}}\right), \end{array}$$

where VideoFeatureExtractor(·) represents the projection function that maps data to feature vectors, θ_vfeat_ denotes its corresponding parameters, $Z_{n}^{\text{vfeat}}\in {\mathbb{R}}^{D\text{vfeat}\times T_{\text{venc}}}$ represents the feature vectors, where *D*_vfeat_ denotes the feature dimension, FC(·) refers to a fully connected layer, and θ_F_C__1__ represents its parameters. The details of these extracted features are described in the “Encoder Configurations” paragraph in Section 4.2.

$Z_{n}^{\text{v}}$ and $Z_{n}^{\text{a}}$ are used as inputs to the CAL with *L* layers,


(20) $$\begin{array}{l} S_{n}^{\text{a}},S_{n}^{\text{v}}=CAL\left(Z_{n}^{\text{a}},Z_{n}^{\text{v}};\theta_{\text{CAL}}\right). \end{array}$$

Subsequently, the interaction among participants was modeled using a global-token sequence. The outputs of the CALs are concatenated to create a participant feature vector $S_{n}\in {\mathbb{R}}^{D\text{model}\times \left(T_{\text{v}}+T_{\text{a}}\right)}$ for each participant. To manage the quadratic complexity of attention, we introduce GTL with *K* layers;


(21) $$\begin{array}{l} S_{n}=\left[S_{n}^{\text{a},L}∥S_{n}^{\text{v},L}\right], \end{array}$$

(22) $$\begin{array}{l} U_{1},\cdots \;,U_{N}={GTL}_{common}\left(S_{1},\cdots \;,S_{N};\theta_{\text{GTLc}}\right). \end{array}$$

Finally, pooling is applied to the output of the final layer of the transformer encoder to obtain the posterior probabilities of the labels as follows:


(23) $$\begin{array}{l} P\left(l_{n^{*}}|X,n^{*},\Theta \right)=softmax\left(FC\left(AP\left(U_{n^{*}};\theta_{\text{AP}}\right);\theta_{\text{F}{\text{C}}_{2}}\right)\right), \end{array}$$

where softmax(·), AP(·), θ_AP_ and θ_FC_2__ represent the softmax-function, attention-pooling-layer, attention-pooling-layer parameters, and fully connected layer parameters, respectively.

The model parameters Θ are optimized by minimizing the cross-entropy loss on the training data D as follows:


(24) $$\begin{array}{l} \Theta =\left\{\theta_{\text{F}{\text{C}}_{1}},\theta_{\text{CAL}},\theta_{\text{GTLc}},\theta_{\text{AP}},\theta_{\text{F}{\text{C}}_{2}}\right\} \end{array}$$

(25) $$\begin{array}{l} L=-\underset{X,n,l_{n}\in D}{\sum }\log P\left(l_{n}|X,n,\Theta \right). \end{array}$$

Notably, the encoder parameters θ_venc_, θ_a_, and θ_vfeat_ are pre-trained and remain frozen during training.

#### 3.3.2 Baseline participant-pairwise bottleneck transformer

To accurately model the interaction between each pair of participants, the baseline PPBT model (Suzuki et al., 2024) defines multiple global token sequences, with each token corresponding to a pair of participants. Specifically, instead of using Equation 22 in the baseline MPBT, PPBT utilizes pairwise GTL to model cross-person interaction.


(26) $$\begin{array}{l} U_{1},\cdots \;,U_{N}={GTL}_{pairwise}\left(S_{1},\cdots \;,S_{N};\theta_{\text{GTLp}}\right). \end{array}$$

The calculation of posterior probabilities from the output of the final layer follows the same process as the baseline MPBT.

### 3.4 Multi-data stream bottleneck transformers

#### 3.4.1 Baseline multi-data stream bottleneck transformer

We introduce the baseline multi-data stream bottleneck transformer (MDSBT). The baseline MDSBT was designed to model interactions across input data streams, i.e., multiple modalities from multiple participants by common global tokens. Instead of modeling cross-modal and cross-person interactions by using Equations 20–22 of a hierarchical MPBT model, the MDSBT models the interactions among all input data streams by GTLs with *K* layers,


(27) $$\begin{array}{l} U_{\left(1,\text{a}\right)},U_{\left(1,\text{v}\right)},\cdots \;,U_{\left(N,\text{a}\right)},U_{\left(N,\text{v}\right)}={GTL}_{common}\left(Z_{1}^{\text{a}},Z_{1}^{\text{v}},\cdots \;,Z_{N}^{\text{a}},Z_{N}^{\text{v}};\theta_{\text{GTLc}}\right). \end{array}$$

Finally, the output variables associated with the target participant are concatenated to form $U_{n^{*}}^{K}$ as follows:


(28) $$\begin{array}{l} U_{n^{*}}=\left[U_{\left(n^{*},\text{a}\right)}∥U_{\left(n^{*},\text{v}\right)}\right]. \end{array}$$

The posterior probabilities of the labels are calculated in the same manner as Equation 23.

#### 3.4.2 Proposed data stream-pairwise bottleneck transformer

The proposed DSPBT introduces pairwise global tokens to the baseline MDSBT. Instead of using Equation 27 of the baseline MDSBT, DSPBT uses pairwise GTL as follows:


(29) $$\begin{array}{l} U_{\left(1,\text{a}\right)},U_{\left(1,\text{v}\right)},\cdots \;,U_{\left(N,\text{a}\right)},U_{\left(N,\text{v}\right)}={GTL}_{pairwise}\left(Z_{1}^{\text{a}},Z_{1}^{\text{v}},\cdots \;,Z_{N}^{\text{a}},Z_{N}^{\text{v}};\theta_{\text{GTLp}}\right). \end{array}$$

Following the process of the baseline MDSBT, the output variables associated with the target participant are concatenated and fed to the classification layer, in the same manner as Equation 23.

## 4 Experiment

### 4.1 Experimental dataset

We used the RoomReader corpus (Reverdy et al., 2022), which comprises multimodal, multiparty conversational interactions where participants engaged in a collaborative online student-tutor scenario designed to elicit spontaneous speech. This dataset was chosen because it reflects realistic online multiparty interactions (4—5 participants), aligning with our aim to capture cross-participant dynamics in engagement. Notably, it has also been utilized in Multipar-T, allowing us to compare results under consistent conditions (Lee et al., 2023). The corpus was processed to separate the audio and video for each participant and synchronize them. The video resolution is 2,560 × 1,440, with a frame rate of 60 fps, and the audio is sampled at 32 kHz with 16-bit quantization. In the experiment, the frame rate was reduced to 8 fps. Although higher frame rates would capture more detailed facial expressions, they would significantly increase GPU memory usage and limit the computing environments in which the model could be trained. The corpus also includes continuous annotations for engagement. The data are labeled every second, and the label at the last second of each clip was used as the target. The labels range from –2 to 2. Instead of a regression task, we defined the task as a four-class classification, where labels in the range [–2, –1] indicate high disengagement (*l* = 1), (–1, 0] indicate low disengagement (*l* = 2), (0, 1] indicate low engagement (*l* = 3), and (1, 2] indicate high engagement (*l* = 4). We trained models on data from 24 groups and tested them on data from 6 groups. Each group consisted of five participants. We split the training and test sets so that the test set did not include any participant in the training set. The number of video clips used for training was 53,192 and 12,756 for testing (a total of 65,948). This was smaller than a previous study (Lee et al., 2023), where 152,614 clips were extracted from the RoomReader corpus, of which 121,305 were allocated for training and 31,309 for testing. This difference was due to our exclusion of clips with errors in face detection and OpenFace-based feature extraction. Specifically, if face region detection or feature extraction failed for even a single frame in a clip, that clip was excluded from the experiments.

### 4.2 Setups

#### 4.2.1 Pre-processing

For video inputs, we detected face regions in each frame using YOLOv3 (Redmon and Farhadi, 2018), which was trained on the Wider Face dataset (Yang et al., 2016). In our experiments, we resized the detected facial regions to a resolution of 128 × 128. While ResNet-50 (He et al., 2016) typically employs inputs with a resolution of 224 × 224, we opted for this lower resolution to reduce GPU memory consumption. This decision was made due to the large volume of data we used, which included both video and audio recordings from five participants. Previous studies have demonstrated that a resolution of 128 × 128 can still achieve sufficiently high accuracy (Touvron et al., 2019), making it a viable choice for our setup. Table 3 shows the frequency of each label in the 8-second video clips from the training and test sets. It is evident that there is a significant class imbalance. To mitigate the effects of class imbalance, we oversampled the infrequent classes to balance the frequency distribution in the training set. We did not use Focal Loss (Lin et al., 2017) because it was not effective under our experimental conditions.

**Table 3:** Frequency of each label in the 8-second video clips from the training and test data.

**Label**	**Train**	**Test**	**Total**	**Ratio (train) (%)**	**Ratio (test) (%)**
High Dis-Eng.	217	45	262	0.4	0.4
Low Dis-Eng.	815	368	1183	1.5	2.9
Low Eng.	10910	1377	12287	20.5	10.8
High Eng.	41250	10966	52216	77.5	86.0

#### 4.2.2 Encoder configurations

We used ResNet-50 (He et al., 2016) as the video encoder. We used the normalized eye-gaze direction, head location, 3D landmark positions, and facial-action units extracted through OpenFace (Baltrušaitis et al., 2016) as the video features. The xlsr-53 features from the final layer were used as the audio features (Conneau et al., 2021).^1^ The feature dimensions were *D*_venc_ = 2,048, *D*_a_ = 1,024, *D*_vfeat_ = 709. The length of each feature was *T*_venc_, *T*_vfeat_ = 64, *T*_a_ = 799. No further feature selection or dimensionality reduction was applied to any of these extracted features, as we opted to retain all available information for engagement estimation.

#### 4.2.3 Methods

We evaluated the baseline CPT (Lee et al., 2023; Kim et al., 2023), MPBT (Section 3.3.1), PPBT (Section 3.3.2), MDSBT (Section 3.4.1), and the proposed DSPBT (Section 3.4.2). To examine the effect of not using a hierarchical structure (“joint modeling”) in MDSBT and DSPBT, we also evaluated MDSBT and DSPBT using a hierarchical structure, i.e., models in which CALs were replaced with GTLs in Figure 1b. We denote these hierarchical models as MDSBT1 and DSPBT1 and the originals as MDSBT2 and DSPBT2. In order to clarify the effect of using multimodal information in each model, not only the multimodal conditions (using video and audio features) but also the single-modal conditions (using only video features) were evaluated. The single-modal condition using only audio features was not evaluated because of the need to estimate the engagement of participants who were silent. Note that in the single-modal condition, MPBT is attributed to the same model structure as MDSBT, and similarly, PPBT is attributed to the same model structure as DSPBT, as there is no cross-modal interaction model.

We describe the setup that was common to all ten models. We set the number of people *N* to 5. For all models, the total number of transformer encoder blocks was unified; *L* and *K* were set to 2 for the hierarchical models (CPT, MPBT, PPBT, MDSBT1, and DSPBT1), while *K* was set to 4 for the joint models (MPBT and PPBT using single modality, MDSBT2, and DSPBT2). We used 8 s of video and audio context information, i.e., *D* = 8. The number of multi-head attention heads was set to 4. We used the rectified linear unit activation function; we trained all models using three seeds to calculate an average score for each experimental condition.

For the conventional CPT, instead of Equation 27 from the baseline model, we used a CAL, where the target participant's features were used as keys and values, and another participant's features were used as queries, following a previous study (Lee et al., 2023). The outputs of the five CPTs were combined, and the model dimension *D*_model_ was set to 256. The batch size was 4, the learning rate was 0.00001, the optimizer was Radam, and early stopping was applied (Liu et al., 2020). To ensure that the results are not due to coincidence along the feature dimension and are used as $U_{n^{*}}$ in Equation 23. For all models, the length of the global tokens *B* was set to 4.

## 5 Results and discussion

Table 4 shows the results (the number of participants *N* = 5). We first compared models that use CALs with those that use GTLs for cross-modal interactions. CPT showed lower accuracy, weighted F1, and macro F1 than all those using all GTL cross-modal interaction models. In the models using common global tokens (MPBT, MDSBT1, and MDSBT2), MDSBT1 and MDSBT2 showed higher accuracy, weighted F1, and macro F1 than MPBT. Similarly, in models using pairwise global tokens (PPBT, DSPBT1, and DSPBT2), DSPBT1 and DSPBT2 showed higher accuracy, weighted F1, and macro F1 than PPBT. On the basis of these results, using GTL for cross-modal interaction is effective.

**Table 4:** Evaluation results (the number of participants *N* = 5; modalities: V, video features; A, audio features).

**Model**	**Cross-modal interaction**	**Cross-person interaction**	**Global tokens**	**Modality**	**All joint engagement classes**			***l* = 1**	***l* = 2**	***l* = 3**	***l* = 4**
					**Accuracy**	**Weighted F1**	**Macro F1**	**F1**	**F1**	**F1**	**F1**
CPT	-	CAL	-	V	0.632	0.675	0.216	0.000	0.016	0.073	0.775
CPT	CAL	CAL	-	V+A	0.646	0.684	0.227	0.000	0.026	0.099	0.783
MPBT	-	GTL	Common	V	0.668	0.693	0.214	0.000	0.002	0.053	0.799
MPBT	CAL	GTL	Common	V+A	0.677	0.707	0.227	0.000	0.035	0.059	0.814
PPBT	-	GTL	Pairwise	V	0.690	0.706	0.217	0.000	0.013	0.038	0.816
PPBT	CAL	GTL	Pairwise	V+A	0.720	0.733	0.236	0.000	0.028	0.073	0.842
MDSBT	-	GTL	Common	V	0.668	0.693	0.214	0.000	0.002	0.053	0.799
MDSBT1	GTL	GTL	Common	V+A	0.689	0.717	0.242	0.000	0.031	0.117	0.819
MDSBT2	GTL (joint)		common	V+A	0.692	0.719	0.249	0.000	0.100	0.073	0.824
DSPBT	-	GTL	Pairwise	V	0.668	0.693	0.214	0.000	0.002	0.053	0.799
DSPBT1	GTL	GTL	Pairwise	V+A	0.735	0.746	0.270	0.000	**0.147**	0.079	0.852
DSPBT2	GTL (joint)		pairwise	V+A	**0.763**	**0.771**	**0.277**	0.000	0.061	**0.176**	**0.872**

Boldface values represent the highest score achieved for each evaluation metric.

Next, the GTL interaction models were compared from the viewpoint of the hierarchical structure. Using common global tokens, we compared MDSBT1 with MDSBT2. MDSBT2 showed higher accuracy, weighted F1, and macro F1. By using pairwise global tokens and GTLs for all interactions, we compared DSPBT1 with DSPBT2, and DSPBT2 showed higher accuracy, weighted F1, and macro F1. On the basis of these results, using a joint model for cross-modal and cross-person interaction modeling is effective.

Additionally, models were compared from the viewpoint of the modality. As in previous studies (Kim et al., 2023), multimodal conditions showed higher accuracy, weighted F1, and macro F1 than the single-modal conditions for each model. Given these results, by incorporating auditory cues–such as the presence or absence of speech, vocal intonation, and speech rhythm–alongside visual information (e.g., facial expression), We can improve the accuracy of engagement estimation for each participant. On the basis of these results, multimodal modeling is effective for the proposed DSPBT as well as the conventional models.

Finally, we examined the effect of adopting the pairwise global tokens for the proposed DSPBT2. We compared MDSBT2 (using common global tokens) and DSPBT2 (using pairwise global tokens), and DSPBT2 showed higher accuracy, weighted F1 and macro F1. The model that uses pairwise global tokens showed the highest accuracy, weighted F1, and macro F1 among all models. On the basis of these results, using pairwise global tokens for the proposed method is effective.

**Figure 5:**
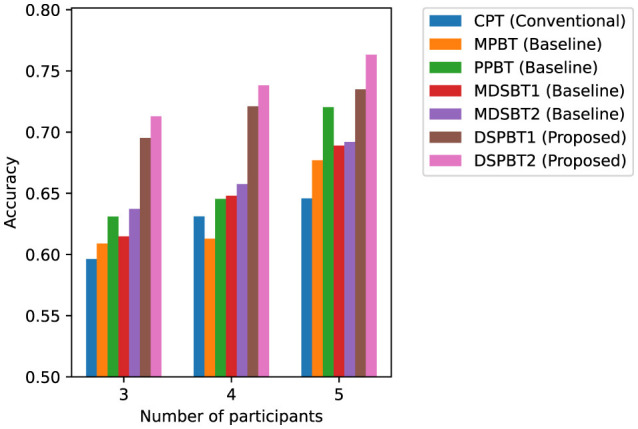
Accuracy across different numbers of participants.

**Figure 6:**
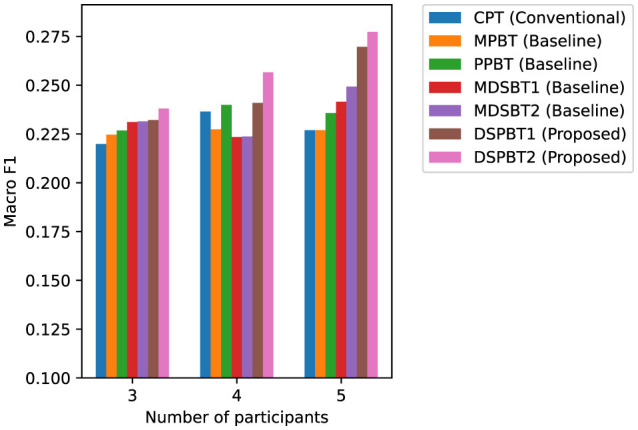
Macro F1 across different numbers of participants.

Figures 5, 6 show the accuracy and macro F1 for different numbers of participants. On the basis of the results of the models using common global tokens (MPBT/MDSBT1) and using pairwise global tokens (PPBT/DSPBT1), using GTLs for cross-modal interaction yielded higher accuracy and macro F1. On the basis of the results of the models using common global tokens (MDSBT1/MDSBT2) and using pairwise global tokens (DSPBT1/DSPBT2), not using a hierarchical structure showed higher accuracy and macro F1. The tendency of DSPBT2 remained consistent regardless of the number of participants. In a two-participant scenario, the difference between “common” and “pairwise” tokens effectively disappears as there is only one pair. As the number of participants decreases, the total number of interactions likewise diminishes, which tends to reduce the model's accuracy.

Regarding the per-class F1-scores, the results for the high disengagement label were not improved. This is likely due to the label-imbalance issue, as shown in Table 3. Addressing this issue requires either using a corpus with balanced labels or further investigating the details of the oversampling techniques.

**Figure 7:**
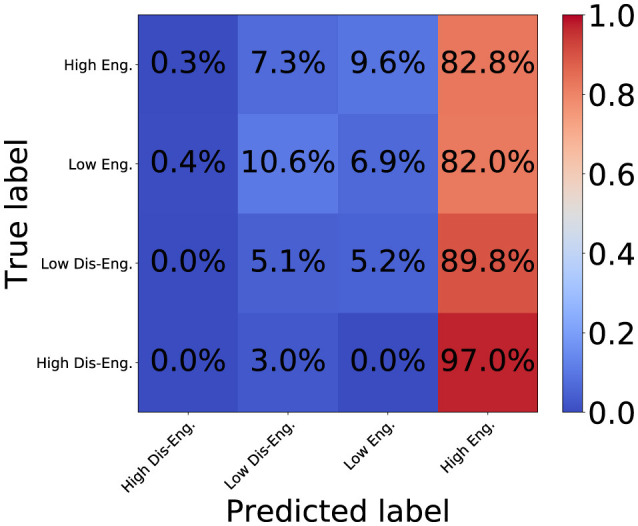
Confusion matrix of baseline PPBT.

**Figure 8:**
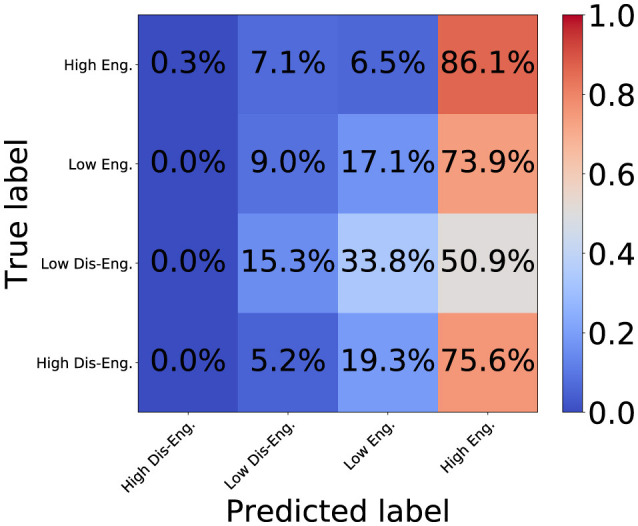
Confusion matrix of DSPBT2.

Figures 7, 8 show the confusion matrix for the proposed DSPBT2 and the baseline PPBT. DSPBT2 showed a higher accuracy than DSPBT1, and the baseline PPBT showed the highest accuracy among the baseline models. DSPBT decreased misclassifications in classifying high disengagement as high engagement. This means that even if it couldn't classify high disengagement, it classified it as low disengagement, which improved the classification performance. DSPBT was more accurate in classifying low disengagement and decreased the misclassification of low disengagement as high engagement. This means that even if it couldn't classify low disengagement, it classified it as low engagement, which improved the classification performance. Low engagement and high engagement can be classified more accurately. In summary, our findings demonstrate that leveraging pairwise global tokens for non-hierarchical cross-modal and cross-person interaction modeling can enhance engagement classification performance. This approach holds promise for multiparty, multimodal tasks in fields such as human–computer interaction, group communication analysis, or social robotics.

The model performs best when each participant is recorded with a front-facing webcam that keeps the face largely inside the frame, mirroring the setup of the RoomReader corpus. Consequently, gallery view video conference recordings or any scenario in which every participant has a dedicated webcam tile are the most suitable inputs. Recordings where faces are small, heavily occluded, or only intermittently visible (e.g., speaker view or wide-angle room cameras) may require additional front facing cues or model retraining to maintain accuracy. By integrating both auditory (e.g., speech presence, vocal intonation, and rhythm) and visual (e.g., facial expression) cues, we achieve more accuracy. Future work will focus on extending these methods to larger and more diverse datasets, as well as exploring strategies to mitigate class imbalance and further improve the classification of minority classes.

## 6 Conclusion

We proposed the data stream-pairwise bottleneck transformer (DSPBT), which uses pairwise global tokens while simultaneously handling both cross-modal and cross-person interactions. Compared with the baseline PPBT, DSPBT not using a hierarchical structure showed better accuracy, weighted F1, and macro F1. These findings confirm our main concept—that unifying cross-modal and cross-person interactions through global token-based transformer effectively reduces redundancy and facilitates more accurate engagement estimation in multiparty settings. Additionally, the method is effective in multimodal conditions. We also showed that this tendency remains consistent regardless of the number of participants from the viewpoint of the multimodal condition.

Building on these findings, our future work will explore more diverse data scenarios, such as varying conversation lengths and many participants, while also addressing class-imbalance challenges through improved data augmentation or tailored loss functions. Ultimately, we aim to make the DSPBT framework more robust, scalable, and adaptable to a broader range of real-world multiparty interactions.

## Data Availability

The original contributions presented in the study are included in the article/supplementary material, further inquiries can be directed to the corresponding author/s.

## Footnotes

^1^ https://huggingface.co/facebook/wav2vec2-large-xlsr-53
